# Functional Interaction between Ribosomal Protein L6 and RbgA during Ribosome Assembly

**DOI:** 10.1371/journal.pgen.1004694

**Published:** 2014-10-16

**Authors:** Megha Gulati, Nikhil Jain, Joseph H. Davis, James R. Williamson, Robert A. Britton

**Affiliations:** 1 Department of Microbiology and Molecular Genetics, Michigan State University, East Lansing, Michigan, United States of America; 2 Department of Integrative Structural and Computational Biology, Department of Chemistry and The Skaggs Institute for Chemical Biology, The Scripps Research Institute, La Jolla, California, United States of America; University of Geneva Medical School, Switzerland

## Abstract

RbgA is an essential GTPase that participates in the assembly of the large ribosomal subunit in *Bacillus subtilis* and its homologs are implicated in mitochondrial and eukaryotic large subunit assembly. How RbgA functions in this process is still poorly understood. To gain insight into the function of RbgA we isolated suppressor mutations that partially restored the growth of an RbgA mutation (RbgA-F6A) that caused a severe growth defect. Analysis of these suppressors identified mutations in *rplF*, encoding ribosomal protein L6. The suppressor strains all accumulated a novel ribosome intermediate that migrates at 44S in sucrose gradients. All of the mutations cluster in a region of L6 that is in close contact with helix 97 of the 23S rRNA. *In vitro* maturation assays indicate that the L6 substitutions allow the defective RbgA-F6A protein to function more effectively in ribosome maturation. Our results suggest that RbgA functions to properly position L6 on the ribosome, prior to the incorporation of L16 and other late assembly proteins.

## Author Summary

Ribosomes are complex macromolecular machines that carry out the essential function of protein synthesis in the cell. The assembly of ribosomal subunits is a multistep process that involves the accurate and timely assembly of 3 rRNA molecules and>50 ribosomal-proteins. In recent years many ribosome assembly factors have been identified in bacterial and eukaryotic cells; however, their precise functions in ribosome biogenesis are poorly understood. We have previously shown that the GTPase RbgA, a protein conserved from bacteria to humans, is essential for ribosome assembly in *Bacillus subtilis*. Here, we show that growth defect caused by a mutation in RbgA is partially suppressed by mutations in ribosomal protein L6. The suppressor strains accumulate novel ribosomal intermediates that appear to suppress the RbgA defect by weakening the interaction of L6 for the ribosome and facilitating RbgA dependent assembly. Our work provides evidence for a functional interaction between ribosome assembly factor RbgA and ribosomal protein L6 during assembly, a function that is likely important for mitochondrial, chloroplast, and eukaryotic ribosome assembly as well.

## Introduction

The assembly of the 30S and 50S ribosomal subunits is a complex and tightly coordinated series of events that consists of the synthesis, processing and modification of 5S, 16S and 23S rRNA and the addition of more than 50 ribosomal proteins (r-proteins) [1], [2], [3]. The *in vitro* reconstitution of a mature 50S subunit has been extensively studied in *Escherichia coli* and the formation of a mature 50S subunit from its constituent r-proteins and rRNA is a multi-step process that requires non-physiological conditions such as high ionic concentration, high temperatures and long incubation times [4], [5], [6], [7]. Relatively fewer studies focused on ribosome assembly in other bacterial species, such as *Geobacillus stearothermophilus*, and these demonstrated that the intermediates formed in this system are different than those in *E. coli*, however similar non-physiological steps are required for formation of a functional ribosomal subunit [5], [8]. Moreover, recent studies have utilized biophysical techniques to study ribosome assembly *in vivo* and demonstrated that assembly of the ribosome subunits is a multistage process that appears to follow multiple parallel pathways in which the accumulation of assembly intermediates identified *in vitro* do not accumulate *in vivo* [9], [10], [11]. The slow kinetics and attenuated efficiency of *in vitro* assembly strongly suggest that assembly factors are involved *in vivo* and indeed, several classes of assembly factors such as GTPases, RNA helicases, RNA modification enzymes and chaperone proteins have been implicated in *in vivo* ribosome assembly in bacterial and eukaryotic cells [2], [12], [13], [14], [15]. However, while studies show that these factors are functionally significant and play a critical role in ribosome assembly, the molecular functions of these factors remain elusive. RbgA (ribosome biogenesis GTPaseA) is an essential GTPase that is required for a late step in assembly of the 50S subunit in *Bacillus subtilis* [16], [17]. RbgA is a widely conserved protein and its eukaryotic homologs such as Mtg1, Lsg1, Nug1 and Nog2 have also been implicated in assembly of the large ribosomal subunit [18], [19], [20], [21]. RbgA depleted cells do not form mature 50S subunits but instead accumulate a 45S complex. Quantitative mass spectrometry analysis of this particle shows that the 45S completely lacks ribosomal proteins L16, L28, and L36 and contains severely reduced amounts of L27, L33, and L35 [16], [22]. Proteins L16 and L27 are crucial components of the peptidyltransferase center in 50S subunit and directly contact the A-site and the P-site respectively [23], [24]. Functional studies have shown that both proteins play a role in stabilization of the peptide bond formation, the positioning of tRNA on their respective sites and are required for optimal functioning of the ribosome [25], [26], [27]. While there have been no reports of deletion of L16, the deletion of L27 in *E. coli* causes a severe growth defect [28]. However, studies in *B. subtilis* indicate that both proteins are essential and deletion mutants could not be obtained for either protein [29]. *In vitro* assembly experiments have demonstrated that incorporation of L16 into the growing complex occurs at a late stage in the assembly process and is accompanied by a large conformational change [30]. In yeast, the RbgA homolog Lsg1 has been proposed to play a role in the incorporation of the L16 homolog Rpl10 into the large ribosomal subunit, suggesting that RbgA and its homologs regulate an evolutionarily conserved step during biogenesis [31], [32]. RbgA has been shown to interact directly with both the 45S complex and the 50S subunits and the GTPase activity of RbgA is enhanced ∼60 fold in the presence of the mature 50S subunit [33]. Mutational analysis of RbgA has shown that a stretch of 15 amino acids in the N-terminal domain, which is largely conserved among all bacterial RbgA homologs as well as eukaryotic homologs, plays a crucial role in this GTPase activity [34]. Mutations that affect GTP hydrolysis result in the accumulation of the 45S complex similar to RbgA depleted cells indicating that GTP hydrolysis plays a key role in maturation of the 50S subunit [34].

To further investigate the role of RbgA in the assembly of the 50S subunit we constructed a *B. subtilis* strain that expressed a mutated RbgA protein that results in a severe growth defect and screened for suppressors that alleviated this growth defect. We isolated and characterized eight independent suppressor strains and found they contained six distinct mutations in the *rplF* gene, which encodes for ribosomal protein L6. Analysis of ribosome assembly in these strains led to discovery of a novel ribosomal intermediate that differs from the 45S complex observed in the parental strain and also in RbgA-depleted cells. We discuss the implications of these results and present a model for the role of RbgA in assembly of the 50S subunit.

## Results

### Construction of a growth-impaired *rbgA* mutant strain and subsequent isolation of suppressor mutants

**Figure 1:**
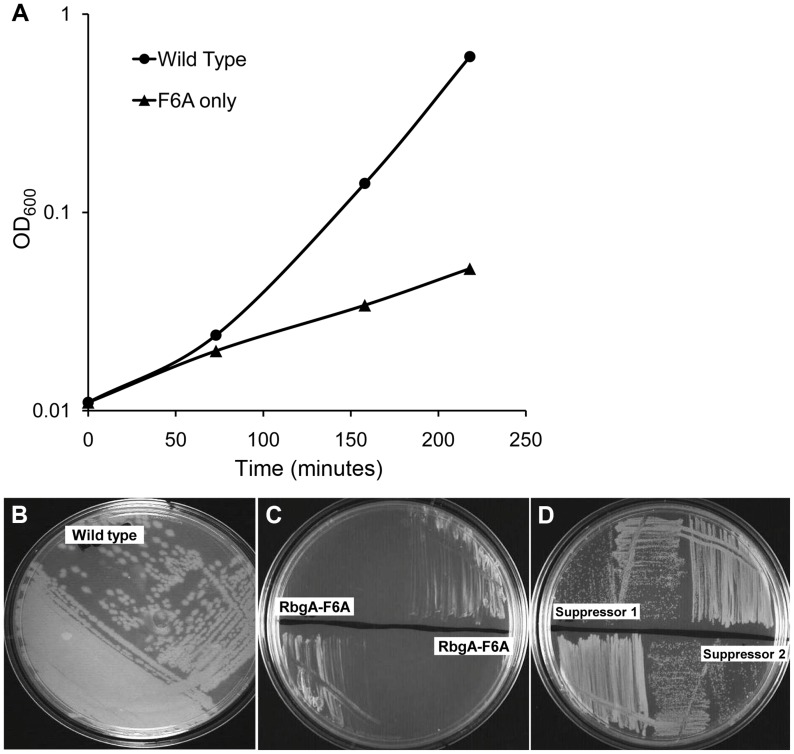
Phenotype of RB1043 (*rbgA*-F6A) and suppressor strains. (A) RbgA-F6A mutation causes a severe growth defect. Strains were grown in LB at 37°C. Strain RB1006, depicted by closed circles, is a control strain with wild type *rbgA* gene under the control of native promoter at *amyE* locus. RB1043, depicted by closed triangles, expresses *rbgA*-F6A gene under the control of native promoter at *amyE* locus. **(B–D)** **Growth of strains on LB plates to compare growth phenotypes.** (B) RB1006 (Wild-type control strain), (C) RB1043 and RB1044. RB1044 is an independent isolate that contains the *rplF*-F6A mutation and has an identical phenotype. (D) Two suppressor strains isolated from RB1043, (RB1051 and RB1068), that partially alleviate the growth defect. All strains were cultured on LB plates at 37°C overnight.

To generate a strain that displayed a strong growth defect that would be amenable to suppressor analysis, we analyzed the phenotypes of over 40 site-directed mutations in the *rbgA* gene [34]. We were interested in identifying substitutions in RbgA that displayed reduced GTPase activity upon association with the ribosome and were still able to bind to the ribosome. One such mutation, *rbgA*-F6A, was identified as meeting both of these criteria. Our results showed that GTPase activity of RbgA-F6A was reduced ∼12 fold, however the mutation did not prevent stable association with the 45S complex and the 50S subunit [34]. Therefore we constructed a strain in which *rbgA*-F6A was the only functional copy of *rbgA* in the cell expecting that cells harboring *rbgA*-F6A would be viable but display reduced growth. To achieve this we constructed strain RB1043 by cloning the *rbgA* gene (containing a mutation that results in a F6A substitution) fused to its native promoter into the plasmid pAS24 and inserted this construct at the *amyE* locus (Table 1). A control strain (RB1006) that contains a wild-type copy of the *rbgA* gene at the *amyE* locus was constructed in similar manner as a control. The native *rbgA* gene was inactivated in both strains by the insertion of a MLS cassette by marker replacement, which led to the complete removal of the native *rbgA* gene. Comparison of the two strains showed that the strain expressing RbgA-F6A (RB1043) was severely growth compromised and exhibited a growth rate ∼7 fold slower than the RB1006 strain (Figure 1A). This severe growth defect was utilized to isolate suppressor mutations that allowed this strain to grow more rapidly.

**Table 1:** Strains used in this study.

Strain	Relevant genotype	Source
RB301	JH 642 P*spank-rbgAcat* pMAP65	[16]
RB419	JH 642 P*spank-infBcat* pMAP65	[16]
RB1006	JH 642 Δ*rbgA*::MLS *amyE*::*rbgA*Spc^r^	This study
RB1032	JH 642 Δ*rbgA*::MLS *amyE*::*rbgA-F6A*Spc^r^	This study
RB1043	JH 642 Δ*rbgA*::MLS *amyE*::*rbgA-F6A*Spc^r^	This study
RB1044	JH 642 Δ*rbgA*::MLS *amyE*::*rbgA-F6A*Spc^r^	This study
RB1051	JH 642 Δ*rbgA*::MLS *amyE*::*rbgA-F6A*Spc^r^ *rplF*-R70P	This study
RB1055	JH 642 Δ*rbgA*::MLS *amyE*::*rbgA-F6A*Spc^r^ *rplF*-R3C	This study
RB1057	JH 642 Δ*rbgA*::MLS *amyE*::*rbgA-F6A*Spc^r^ *rplF*-H66L	This study
RB1059	JH 642 Δ*rbgA*::MLS *amyE*::*rbgA-F6A*Spc^r^ *rplF*-H66L	This study
RB1061	JH 642 Δ*rbgA*::MLS *amyE*::*rbgA-F6A*Spc^r^ *rplF*-H66L	This study
RB1063	JH 642 Δ*rbgA*::MLS *amyE*::*rbgA-F6A*Spc^r^ *rplF*-G5C	This study
Rb1065	JH 642 Δ*rbgA*::MLS *amyE*::*rbgA-F6A*Spc^r^ *rplF*-G5S	This study
RB1068	JH 642 Δ*rbgA*::MLS *amyE*::*rbgA-F6A*Spc^r^ *rplF*-T68R	This study
RB1095	JH 642 *adk*::Cm P*_spank_ map*	This study
RB1102	JH 642 Δ*rbgA*::MLS *amyE*::*rbgA-F6A*Spc^r^ *rplF*-R70P *adk*::Cm P*_spank_ map*	This study
RB1103	JH 642 Δ*rbgA*::MLS *amyE*::*rbgA-F6A*Spc^r^ *rplF*-R3C *adk*::Cm P*_spank_ map*	This study
RB1106	JH 642 Δ*rbgA*::MLS *amyE*::*rbgA-F6A*Spc^r^ *rplF*-G5S *adk*::Cm P*_spank_ map*	This study
RB1107	JH 642 Δ*rbgA*::MLS *amyE*::*rbgA-F6A*Spc^r^ *rplF*-T68R *adk*::Cm P*_spank_ map*	This study
RB1117	*rplF*-R70P *adk*::Cm P*_spank_ map*	This study
RB1118	*rplF*-R3C *adk*::Cm P*_spank_ map*	This study
RB1121	*rplF*-G5S *adk*::Cm P*_spank_ map*	This study
RB1122	*rplF*-T68R *adk*::Cm P*_spank_ map*	This study
RB1123	JH 642 *rplF*-R70P	This study
RB1125	JH 642 *rplF*-R3C	This study
RB1131	JH 642 *rplF*-G5S	This study
RB1133	JH 642 *rplF*-T68R	This study

To isolate independent, spontaneous suppressor mutations we inoculated a single colony of the RB1043 (*rbgA*-F6A) strain per flask into a total of 50 flasks and isolated suppressors that exhibited faster growth at 37°C (only one per flask). We identified eight independent suppressor strains that partially alleviated the growth defect of RB1043 (Figure 1 and Table 2). Individual suppressors were grown in liquid medium and their growth rates were compared to the parental RB1043 strain and the control strain RB1006. The wild-type control strain RB1006 and the parental RB1043 strains exhibited a doubling time of 23 minutes and 173 minutes, respectively, whereas the growth rate of the suppressor strains ranged from 46 to 77 minutes (Table 2). Next, we sequenced the *rbgA*-F6A gene to check for reversion mutations and found that all eight strains lacked intragenic suppressor mutations. We then proceeded to backcross each suppressor strain with the wild-type RB247 strain and inactivated the native *rbgA* gene. The reappearance of RB1043 phenotype (∼7-fold increase in doubling time) in each backcrossed strain indicated that the suppressor mutation was unlinked to the *rbgA*-F6A mutation.

**Table 2:** Suppressor mutations in ribosomal protein L6.

		*rplF* gene	L6 protein	
Suppressor/Strain	Strain number	codon change	Amino acid change	Doubling time
Control strain	RB1006	Wild type	none	23±1
Suppressor 1	RB1055	cgt to tgt	R3C	69±1
Suppressor 2	RB1063	ggt to tgt	G5C	77±1
Suppressor 3	RB1065	ggt to agt	G5S	52±10
Suppressor 4, 5 and 6	RB1057, RB1059, RB1061	cat to ctt	H66L	66±10
Suppressor 7	RB1068	acg to agg	T68R	49±5
Suppressor 8	RB1051	cgc to ccc	R70P	46±4
Parental strain (RbgA-F6A)	RB1043	Wild type	none	173±5

### Suppressor mutations localize to the *rplF* gene, which encodes the ribosomal protein L6

**Figure 2:**
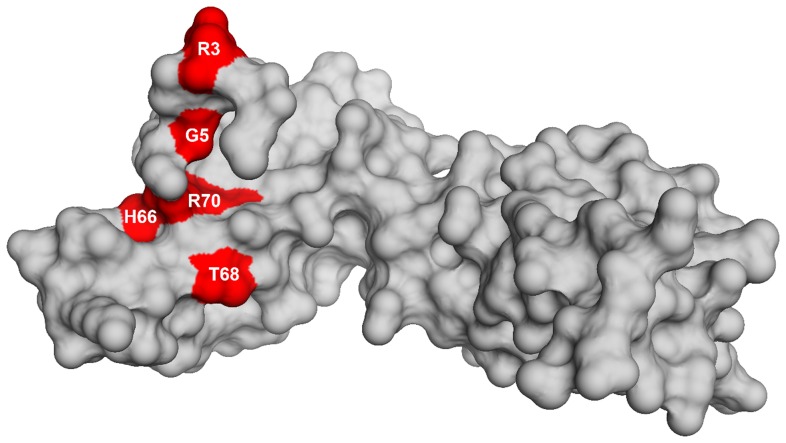
Homology model of L6 protein highlighting the substitutions that suppress the defect in RbgA. Homology model of L6 protein of *B. subtilis* is shown as a surface representation in grey. The residues that are altered in L6 are highlighted in red with the corresponding amino acid labeled.

To identify the genetic changes responsible for the partial suppression of the growth defect we obtained the whole genome sequence of all eight suppressor strains, RB247 (wild-type background) and the parental RB1043. The sequence reads from the parental RB1043 (*rbgA*-F6A) strain were compared with each suppressor strain sequentially. After accounting for mutations that have arisen in our genetic background or were sequencing errors in the original *B. subtilis* sequencing project [35], we found that each suppressor strain bore a single point mutation in the ribosomal protein L6 encoding gene *rplF* gene. Three suppressor strains had the same mutation (Table 2) and thus we obtained six unique suppressor mutations that caused single amino acid substitutions in L6; R3C, G5C, G5S, H66L (3 isolates), T68R and R70P. Alignment of L6 proteins from phylogenetically diverse bacteria indicates that these residues are conserved in bacterial L6 proteins, with T68 demonstrating the most conservation when compared to L6 homologs from archaea and eukaryotes (Figure S1). We constructed a homology model of the *B. subtilis* L6 protein based on the structure of the L6 protein from *Geobacillus stearothermophilus* and mapped the suppressor mutations onto the modeled structure of the protein. Our analysis shows that all of the six suppressor substitutions reside in close vicinity in the protein structure (Figure 2) and are contained within the N-terminal structural domain.

### Suppressor strains accumulate a novel ribosomal intermediate that is distinct from the 45S particle

**Figure 3:**
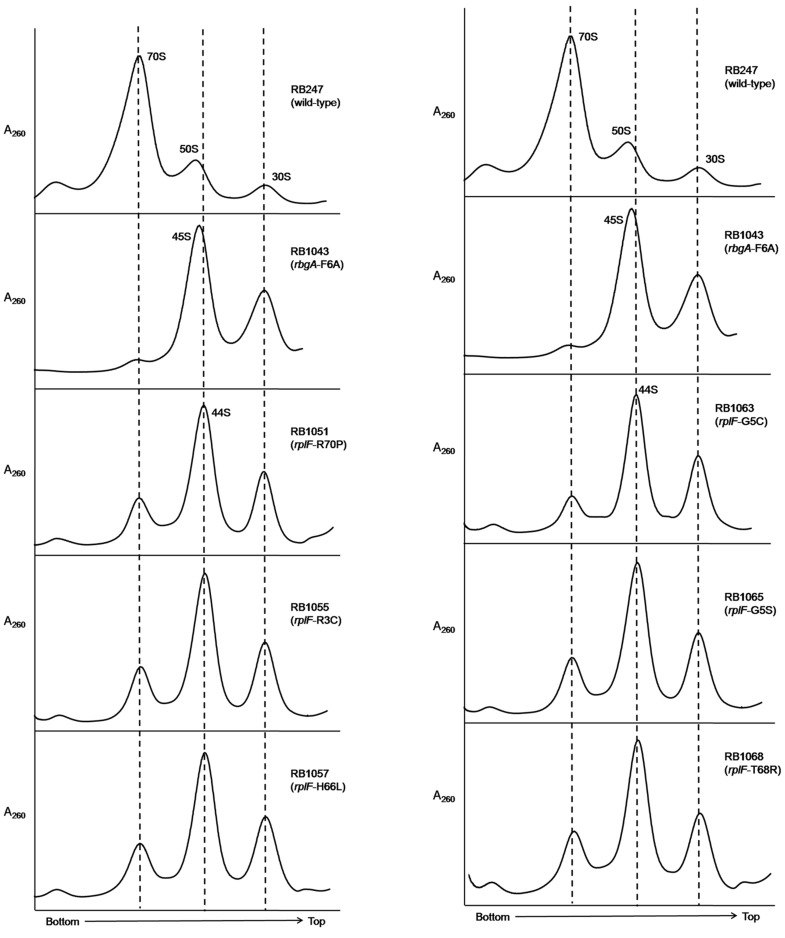
Analysis of ribosome assembly in a L6 suppressor strains. The ribosome profiles of *rbgA*-F6A suppressor strains show an accumulation of a novel 44S complex. Ribosome profiles were analyzed from RB247 (wild-type cells), RB1043 (RbgA-F6A mutant), RB1051 (*rbgA*-F6A, *rplF*-R70P), RB1055 (*rbgA*-F6A, *rplF*-R3C), RB1057 (*rbgA*-F6A, *rplF*-H66L), RB1063 (*rbgA*-F6A, *rplF*-G5C), RB1065 (*rbgA*-F6A, *rplF*-G5S) and RB1068 (*rbgA*-F6A, *rplF*-T68R). Profiles were generated by sucrose density gradient centrifugation. Dashed lines indicate the migration of the 70S, 44S and the 30S subunits in the gradient.

To assess the status of ribosome assembly in the suppressor strains, we analyzed the ribosome profiles using 10–25% sucrose density gradients. Our results showed that all of the suppressor strains accumulated a novel ribosomal intermediate that migrated at ∼44S and was distinct from the 45S complex that accumulates in RbgA-depleted cells and RB1043 strain expressing RbgA-F6A (Figure 3). In addition, each suppressor strain exhibited an increased 70S ribosome peak compared to RB1043, corresponding to the increased growth rate of the suppressor strains.

### Intermediates from suppressor strains lack specific late-binding r-proteins

Given the changes in these suppressor strains' intermediate particle migration patterns, we set out to identify compositional differences between the 44S and 45S intermediates by isolating the particles on a sucrose gradient and measuring their r-protein composition using quantitative mass spectrometry (qMS). As described in materials and methods, our SILAC-like approach resulted in multiple independent peptide measurements for the ribosomal proteins. Additionally, standard curves measured with our technique exhibited a linear dose-response between 0.1 and 1.6 r-protein equivalents (Figure S2), providing confidence in the precision of the approach.

**Figure 4:**
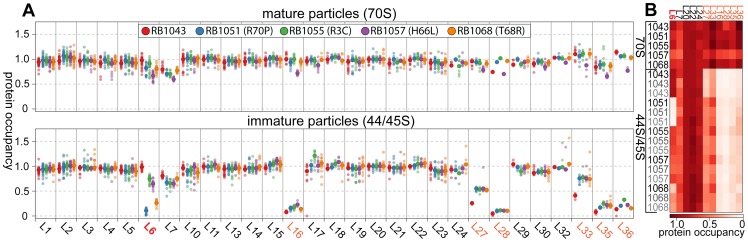
Protein composition of 44S and 45S particles. (A) Ribosomal protein occupancy in 70S (top) or 44S/45S (bottom) samples. Protein occupancy is reported as the corrected ^14^N/^15^N ratio. To account for differences in the amount of sample analyzed, for each peptide, this ratio is normalized to the median calculated ratio for L20, a protein bound stoichiometrically in all samples. Each sample bore the RbgA F6A mutation as well as additional L6 mutations as noted in the legend. Semitransparent dots represent individual peptide measurements. The median value is indicated with a larger opaque marker. Protein L6 is highlighted in red. Proteins significantly depleted are colored orange. **(B) Replicate analysis of selected ribosomal protein occupancy.** Median protein occupancy values are reported as a heat map for either 70S or 44S/45S samples, which were analyzed in triplicate using independent biological samples. Protein occupancy ranging from 0 to 1.1 is colored white to red. Row labels are color coded by replicate (black to grey). Column labels are colored as described above.

Whereas most proteins were present at stoichiometric levels in the intermediates, we found that these particles were severely lacking in proteins L16, L28, L35, and L36 (occupancy<0.4), and were significantly depleted of proteins L27 and L33 (occupancy<0.8) (Figure 4A). These latter depletion effects were more pronounced in the parental RB1043 strain than in any of the suppressor strains, suggesting that these proteins are more efficiently incorporated as a result of the suppressor mutations. Protein L6 showed the greatest variability in protein occupancy across the strains with the parental RB1043 exhibiting full protein incorporation whereas the suppressors RB1051 and RB1068 largely lacked L6 (occupancy<0.3). Interestingly, the intermediates from suppressor strains RB1055 and RB1057 showed more mild L6 depletion effects (occupancy ∼0.8 and 0.6, respectively) despite migrating similarly to the other suppressor strain intermediates. This result suggested that the difference in migration between the 44S and the 45S intermediates arouse from conformational changes in the intermediates and was not a direct result of the extent of L6 incorporation. Finally, we measured protein occupancy in these intermediates from three independent biological replicates and consistently identified that the aforementioned proteins were depleted from the intermediate particles (Figure 4B), confirming the significance of the observed effects.

We then determined the protein composition of each 70S particle from these strains to test whether the protein depletion effects we observed in the intermediate particles persisted into the 70S fractions. The depletion effects observed in the intermediates were largely absent from the mature particles, with only strain RB1057 exhibiting relatively mild occupancy defects (occupancy>0.7) in proteins L6, L16, L27, L28, L35, and L36 (Figure 4A, B). Whether these subtle effects result from instability of the RB1057 70S particles during purification or from a subpopulation of particles that lack these r-proteins in vivo remains to be investigated. In all other strains tested, each r-protein was present at equal stoichiometry with the exception of the rapidly exchanging protein L7/L12 [36], which likely dissociates during particle purification. Taken together, these data affirmed that the mutant L6 proteins, along with the full complement of other large subunit proteins, are integrated during the late assembly stages of the 70S particles.

### 44S intermediate particles only mildly stimulate RbgA GTPase activity

To test if the L6 levels in the complex influenced the GTPase activity of RbgA, we incubated RbgA with each 44S intermediate and measured the rate of GTP hydrolysis. Our results show that GTPase activity of RbgA is stimulated ∼4–6 fold in the presence of the each 44S intermediate, with no correlation between the hydrolysis rate and L6 occupancy (Figure S3). This increase in GTPase activity is similar to the fold change observed in the presence of the 45S complex and highly reduced compared with the ∼60 fold stimulation observed in the presence of the mature 50S subunit [33].

### *rplF* mutations do not impair growth but are partially defective in ribosome subunit joining

We were interested in studying the effects of the alterations in ribosomal protein L6 on cell growth and ribosome assembly in an otherwise wild-type background. To do this we created strains in which the *rplF* mutations were linked to an antibiotic resistance marker and moved into the wild-type background RB247. Once each mutation was transferred into a wild-type background, the antibiotic resistance marker was easily removed by passage on media without selection, resulting in strains that only contained mutations in *rplF* (see materials and methods for details). We successfully constructed strains in which mutations in *rplF* resulting in the R3C (RB1125), G5S (RB1131), T68R (RB1133), and R70P (RB1123) substitutions were the only alterations in the chromosome (Table 1). Each L6 mutant strain's growth rate was indistinguishable from the congenic wild-type RB247 strain, demonstrating that the partial suppression of the RbgA-F6A growth phenotype was not due to an impairment of growth due to defects in L6.

**Figure 5:**
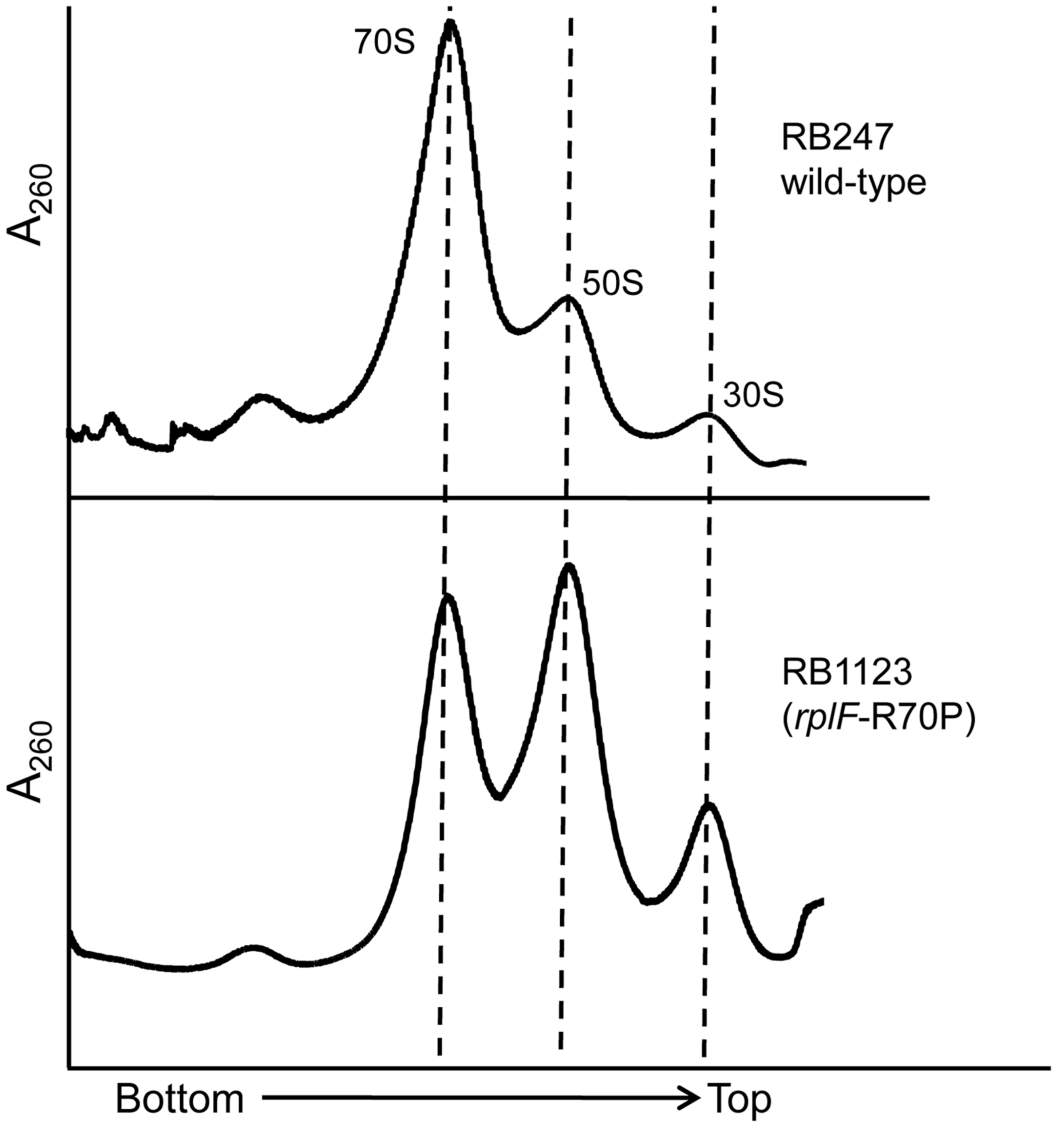
Mutations in L6 protein affect subunit joining/interaction. Ribosome profiles of strains expressing mutated L6 protein [representative strain RB1123 (*rplF*-R70P)]. The X-axis indicates the direction of the profiles from the bottom of the gradient (25%) to the top of the gradient (10%). The Y-axis depicts absorbance at 260 nm, which is equivalent for both plots depicted. Dashed lines indicate the migration of the 70S, 50S and the 30S complexes in the gradient.

Although the *rplF* mutations did not have an effect on cell growth, we were interested to identify if they had any impact on ribosome maturation. Ribosome profiling of strains RB1123, RB1125, RB1131 and RB1133 through 10–25% sucrose gradients was performed and in each case L6 substitutions resulted in abnormal ribosome profiles (Figure 5, S4). The mutants had increased levels of individual ribosomal subunits when compared to wild-type cells, indicating that the L6 substitutions impacted subunit joining or maintenance of 70S ribosome stability. We further analyzed the 50S subunits that accumulated in these strains and found that both ribosomal proteins L6 and L16 were present in levels similar to wild-type 50S subunits indicating that these substitutions in RplF (R3C, G5S, T68R and R70P) do not impact the association of L6 or L16 with the 50S subunit in the context of wild-type RbgA.

### The 44S particle can be matured into a 50S subunit *in vitro*

**Figure 6:**
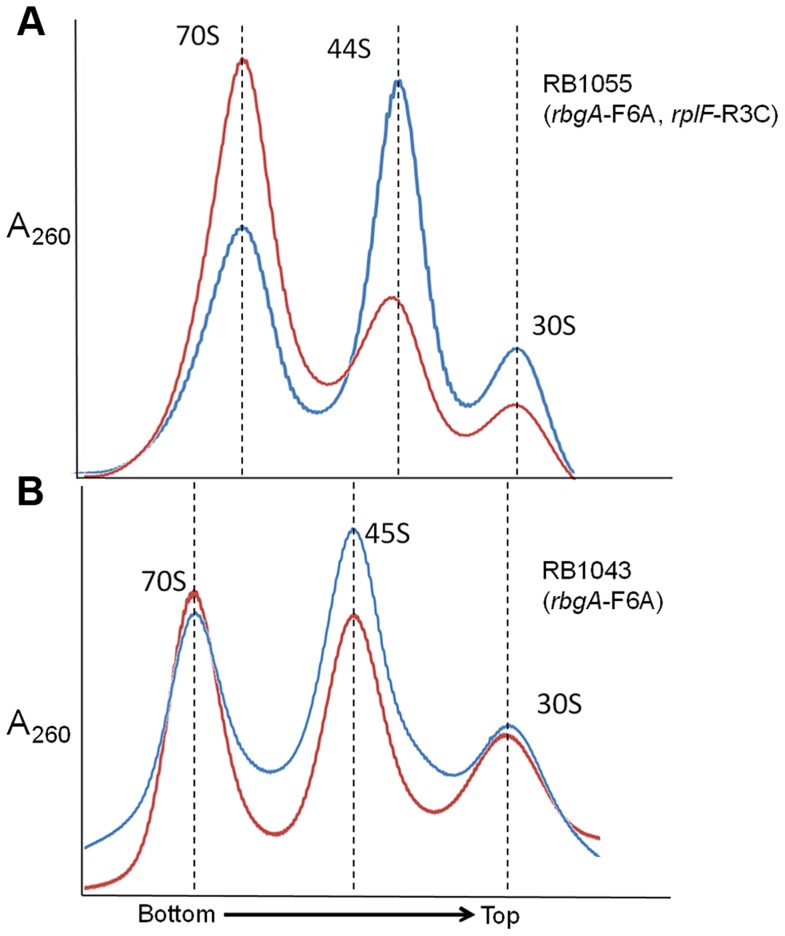
*In vitro* maturation of large subunit intermediates. **(A) *in vitro* maturation of 44S intermediate from RB1055.** Ribosome profile from cell lysate of strain RB1055 expressing mutated L6 protein (R3C) and RbgA-F6A protein after incubation at 0°C (blue) and 37°C (red) for 60 minutes. **(B)** ***in vitro*** **maturation of 45S intermediate from RB1043.** Ribosome profiles from cell lysate of strain RB1043 expressing RbgA-F6A protein and wild-type L6 protein after incubation at 0°C (blue) or 37°C (red) for 60 minutes. The X-axis indicates the direction of the profiles from the bottom of the gradient (43%) to the top of the gradient (18%). The Y-axis depicts absorbance at 260 nm, which is equivalent for both plots depicted. Dashed lines indicate the migration of the 70S, 50S, 44S and the 30S complexes in the gradient.

One possible mechanism for how L6 substitutions may suppress the RbgA-F6A defect is that 44S particles may be more easily matured into 50S subunits than 45S particles. To address this possibility we concentrated cellular lysates from RB1043 (*rbgA*-F6A) and RB1055 (*rbgA*-F6A, *rplF*-R3C) and incubated them for 1 hour at either 37°C or, as a negative control, at 0°C. After incubation, these lysates were centrifuged over 18–43% sucrose gradients in the presence of 20 mM Mg^2+^ (to facilitate mature subunit joining since L6 mutants show subunit association defects in 10 mM Mg^2+^, see Figure 5) [37]. After incubation of the RB1055 lysate at 37°C, we found that many of the 44S particles in the RB1055 lysate were converted into 50S subunits that subsequently partnered with 30S subunits to form 70S ribosomes (Figure 6A). Indeed, 70S ribosomes showed a more than 100% increase during 37°C incubation with a concomitant decrease in 44S and 30S subunits. The RB1043 lysate yielded a much lower level of 70S formation, with only a 10% increase in 70S ribosomes when incubated at 37°C and similar small reductions in 45S and 30S subunits (Figure 6B). These data were consistent with the hypothesis that 44S particles mature into 50S subunits more quickly than 45S particles in vitro.

### Mutant RbgA strains are depleted of late binding r-proteins

**Figure 7:**
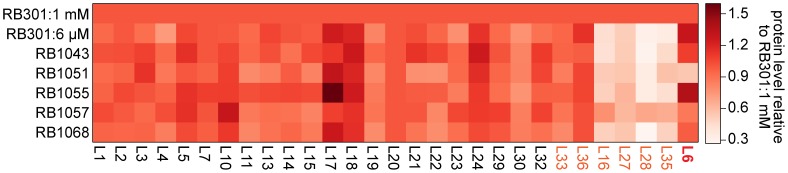
Whole cell protein abundance. A heat map depicting the normalized ribosomal protein abundance in various RbgA perturbation strains. The rows correspond to strains induced RbgA expression (RB301:1 mM), RbgA depletion (RB301:6 µM), RbgA-F6A (RB1043), and RbgA-F6A with suppressor mutations (RB1051:R70P, RB1055:RC3, RB1057:H66L, RB1068:T68R). To facilitate comparison between conditions, each protein's abundance is normalized to that of L20 and then to the protein's level in strain RB301:1 mM. Protein abundance is colored from white (0.3) to red (1.5). Proteins depleted from the 44/45S particles are labeled in orange. Protein L6 is highlighted in red.

Previously, we found that cells depleted of RbgA had very small precursor pools for the r-proteins L16, L27, L28, and L35 [22]. This result suggested that upon RbgA depletion the cell down-regulated synthesis of these proteins through an as-yet uncharacterized mechanism, resulting in very low cytosolic levels of free (unbound) copies of these proteins. To determine if these suppressor strains also lacked free equivalents of these proteins, we directly measured their whole-cell stoichiometry using qMS, specifically, an isotope-label based selective reaction monitoring protocol (SRM) focused on ribosomal peptides (see materials and methods). As predicted by our precursor pool measurements, we found depressed whole-cell protein levels for L16, L27, L28, and L35 in strain RB301 starved for RbgA (Figure 7, S5; RB301:6 µM). In contrast, cells grown with near wild-type levels of this factor exhibited significantly greater levels of each of these proteins (Figure 7, S5; RB301:1 mM IPTG). Assays with strain RB1043, and the L6 suppressors also revealed significant cellular depletion of this set of proteins, indicating that this same regulatory mechanism is activated the RbgA F6A strain and the suppressor mutants. In contrast, protein L36, which is also depleted from the intermediate particles, was abundant in the whole cell lysate. This result is consistent with unregulated synthesis or degradation that results in significant free (unbound) quantities of protein L36.

To determine if the suppressor mutations affected translation or degradation of protein L6, we next inspected the abundance of this protein in each lysate. Interestingly, its level varied greatly between the different suppressor strains with RB1055 lysates bearing ∼2.5 more L6 than those of RB1051. Indeed, the low cellular L6 abundance in RB1051 may explain the low protein occupancy observed in its 44S intermediate particle. Notably, however, with the exception of strain RB1051, L6 abundance in the whole cell lysates correlated poorly with occupancy in the intermediate particles (Pearson's r = 0.37). This result argues that the variable L6 occupancy observed in the 44S particles is not strictly a result of altered translation or degradation of the mutant proteins but, rather, results at least in part from effects of the mutations on protein incorporation or complex stability.

### *In vitro* maturation of 44S particles incorporates a full complement of r-proteins

**Figure 8:**
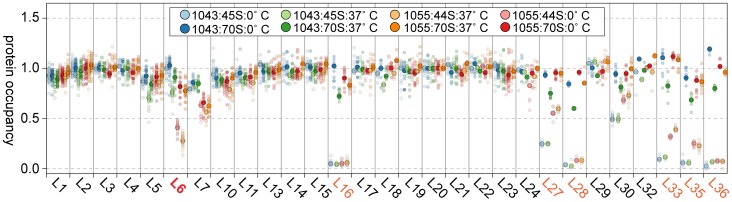
Composition of in vitro matured particles. Protein occupancy in 44/45S and 70S samples normalized to that of L20. Samples are colored in pairs with the 44/45S intermediate in a lighter shade than the 70S particle. Samples from strain 1043 (RbgA-F6A) are on the left (blue, green), those from strain 1055 (RbgA-F6A, L6-RC3) are on the right (orange, red). Semitransparent dots signify unique peptide measurements. The median value is denoted with a larger opaque marker. Protein L6 is highlighted in red. Proteins significantly depleted are colored orange.

Given the depressed levels of proteins L16, L27, L28, and L35 in the suppressor strain lysates, we were curious if these proteins were in fact incorporated into the 70S particles during our in vitro maturation assays. Using qMS, we measured the protein composition of both the precursor 44/45S and product 70S particles from RB1043 and the suppressor strain RB1055 at 0 or 37°C. Whereas the precursor particles were depleted of L16, L27, L28, L33, L35, and L36 (Figure 8; light circles), we found that the 70S particles contained nearly stoichiometric quantities of each of these proteins (Figure 8; dark circles). Although the extent of maturation in strain RB1055 showed a strong temperature-dependence (Figure 6A), the protein occupancy patterns were effectively temperature-independent indicating that the 70S particles formed during our 37°C in vitro maturation assay were indistinguishable from those formed in vivo and maintained during the 0°C incubation (Figure 8; dark orange, dark red).

## Discussion

We provide evidence that mutations causing substitutions in the N-terminal domain of L6 can suppress ribosome assembly defects associated with a mutation that impairs the function of RbgA. Formally, these L6 suppressor mutations could be acting to facilitate assembly either by allowing the defective RbgA-F6A protein to function more effectively in assembly or by allowing ribosomes to assemble in an RbgA-independent pathway. In testing for an RbgA-independent assembly pathway, we repeatedly attempted to generate an *rbgA* null mutation in the background of several of the *rplF* suppressor mutations but were unsuccessful. If the L6 substitutions were able to completely bypass the need for RbgA during maturation then we should have easily isolated null mutations in *rbgA* in the *rplF* mutant backgrounds. Additionally, if significant flux where flowing through an RbgA-independent assembly pathway in these mutant L6 strains, we would expect to find 44S particles in strains bearing wild-type RbgA and L6 mutations. Instead, we only identified 50S particles. Critically, these 44S particles, which require RbgA-F6A, can be matured into 70S ribosomes in vitro. Taken together, we propose that the partial suppression of growth and ribosome assembly defects observed are not due to the L6 substitutions completely bypassing the requirement for RbgA in the cell, but rather, that RbgA function is still required for maturation.

**Figure 9:**
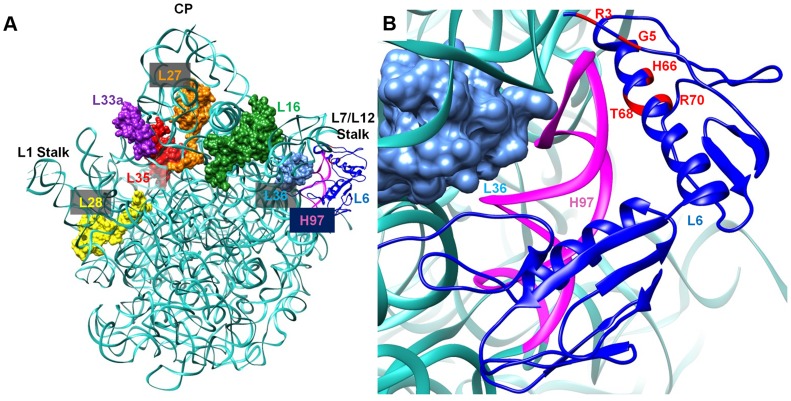
Interaction between L6 protein and the 50S ribosomal subunit. **A**. Crystal structure of 50S subunit from *E. coli* (PDB ID:2AW4) with the position of L6 indicated in blue. The location of late binding ribosomal proteins that are missing or highly reduced in the 45S particle are also highlighted (L16 (green), L28 (yellow) and L36 (cyan)} or highly reduced {L27 (orange), L33a (purple), L35 (red). **B**. L6 (blue) binding region including helix 97 (colored magenta) is shown in a magnified view and the residues mutated in suppressor strains are colored in red at the N terminal of L6 protein.

L6 is a two-domain protein that is located on the L7/L12 side of the 50S subunit and forms an L-like structure that appears to bridge between the front and the back of the subunit (Figure 9) [38], [39]. The N-terminus of the protein interacts with helix 97 (h97) of the 23S rRNA, while the C-terminus of L6 interacts with the sarcin/ricin loop (SRL) [38], [40], [41]. All of the L6 substitutions that suppress the RbgA-F6A defect map to a small region in the N-terminus of the protein and, in some cases, appear to disrupt direct interactions between L6 and h97 (Figure 9). Although some of the suppressor mutations cause L6 to unstably associate with the 44S intermediate (T68R, R70P), the ability to suppress the RbgA-F6A defect does not seem to correlate with L6 binding as 44S particles isolated from the other suppressors contain near wild-type levels of L6 (Figure 4B). The consequence of the L6 substitutions in a wild-type background appears to be at the level of 70S stability. Individual 50S subunits that contain mutant L6 proteins appear to have normal amounts of both L6 and L16, indicating that once matured, these proteins are stably incorporated. However, clearly there is some disruption of 50S subunit structure that causes decreased stability of 70S ribosomes. This is possibly due to improper positioning of the intersubunit bridge helix 89, which is located between and makes direct contacts with L16 and L6.

What effect might mutations in L6 have in suppressing ribosome assembly defects associated with reduced function of RbgA? L6 binds prior to L16 and has been implicated in setting up the binding site for L16 [4], [42]. In *E. coli*, the expression of ribosomes that are deleted for the SRL, which interacts with the C terminus of L6, are dominant-lethal and result in the accumulation of 50S subunits that lack L16 [43]. Lancaster *et al.* propose that L6 binds to the assembling subunit via initial interactions between the N-terminus of L6 and h97, which then results in the subsequent assembly of the functional core of the 50S subunit [43]. This includes the formation of several key interactions between h97, h42, h89, h91, and h95, which are predicted to be initiated by the binding of L6 with h97. When the SRL is deleted these interactions are disrupted and the L16 binding site, along with other functional regions of the large subunit, are improperly assembled and non-functional.

**Figure 10:**
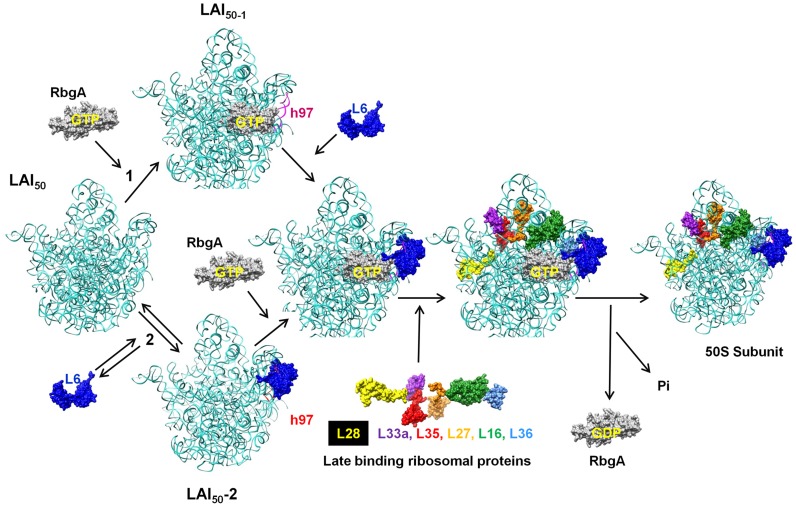
Proposed model for the role of RbgA in promoting late-stage large ribosome subunit assembly. A late assembly intermediate (LAI_50-1_) can proceed via two different pathways. Pathway 1 posits that RbgA binds prior to L6 (LAI_50-2_) while pathway 2 indicates L6 binds prior to RbgA (LAI_50-3_). When bound together (LAI_50-4_), RbgA facilitates proper interaction between L6 and the maturing ribosome, which triggers the incorporation of late ribosomal proteins. Once proper incorporation occurs, RbgA leaves the complex. The role of GTP hydrolysis in the assembly process is discussed in the text.

While the precise role that RbgA plays during ribosome assembly is still unknown, the identification of the second-site suppressors in L6 supports a model in which RbgA participates in facilitating the correct association of L6 with the ribosome to allow the subsequent maturation events to take place (Figure 10). Recent studies have postulated that ribosomal subunits can be formed via multiple parallel pathways [44]. We suspect that the large subunit pathways converge on a late assembly intermediate (LAI_50-1_) and GTPases, such as RbgA, act on LAI_50-1_ to complete maturation. We envision two scenarios in which RbgA could act on LAI_50-1_ to facilitate maturation. In scenario 1, RbgA binds to an undefined late assembly intermediate (LAI_50-2_), and promotes the rearrangement or movement of helix 97 to facilitate the correct incorporation of L6. In scenario 2, L6 binds to the ribosome prior to RbgA (resulting LAI_50-3_, equivalent to the 45S complex) in an unproductive interaction and the role of RbgA binding is to promote the correct interaction of L6 with the helix 97 [43]. We suspect in RbgA mutants the interaction of L6 with LAI_50-2_ is reversible and the suppressor mutations in L6 enhance this reversible step by weakening the interaction with the ribosome. Recently, we have shown that the 45S particle is not a dead end particle and can be fully matured into a 50S particle in vivo. The fact that L6 is not fully visible in the cryo-EM structures of the 45S complex provides support that L6 is not in its proper conformation. In both scenarios, correct positioning of L6 and h97 allows for proteins L16, L27, L28, L33, L35, and L36 to be stably incorporated into the large subunit. Once RbgA senses that incorporation of these proteins has taken place GTP hydrolysis occurs, a final maturation event takes place, and RbgA leaves the subunit. Because we have not been able to isolate RbgA mutants that are deficient in GTPase activity that form 50S subunits, we predict that the GTP hydrolysis plays a dual role in both promoting conformational changes in the ribosome while also resulting in RbgA dissociation. Support for this latter step stems from the fact that 50S subunits lacking only ribosomal proteins L16 and L28 do not stimulate the GTPase activity to levels observed with wild-type 50S subunits [22].

Although we do not know the order of binding of L6 and RbgA, in both scenarios the proposed role of RbgA is to properly position L6 and helix 97 to facilitate assembly. This interaction between L6 and h97 is evolutionarily conserved (see Figure S6) and, given that RbgA homologs are present in archaea and eukaryotes, the role of RbgA proteins in ribosome assembly is likely to be conserved as well. Thus it appears that in small subunit and large subunit ribosome biogenesis, one function of assembly factors is to prevent binding of late binding ribosomal proteins until the subunit is ready to receive them [22], [45], [46]. Whether or not these potential checkpoints are related to quality control mechanisms that insure only functional ribosomes enter into translation remains to be seen [22], [47]. Interestingly, *E. coli* and many other proteobacteria lack RbgA, a function that was present in the last common ancestor and subsequently lost in this lineage of bacteria. We are currently using a comparative genomics approach to identify differences between *E. coli* and *B. subtilis* ribosomes in an attempt to further localize the precise site and mechanism of RbgA function.

## Materials and Methods

### Growth conditions

All strains were grown at 37°C in LB medium and cultures were shaken at 250 rpm. Antibiotics were added at the following concentrations when required: chloramphenicol (5 µg/ml), erythromycin (5 µg/ml), lincomycin (12.5 µg/ml), spectinomycin (100 µg/ml) and ampicillin (100 µgml). IPTG was added to a final concentration of 1 mM when required for strain growth.

### Plasmids

Plasmid pMA1 was derived from pSWEET, an *amyE* insertion vector with a chloramphenicol resistance cassette, by placing the *rbgA* gene under the control of a xylose inducible promoter. Plasmid pAS24, an *amyE* insertion vector with a spectinomycin resistance, was used to construct pMG28 by inserting a wild-type copy of *rbgA* under the control of its native promoter. Plasmid pMG29 bearing a F6A mutation in the *rbgA* gene (accomplished by a TTC to GCC codon change) was constructed from pMG28 using the QuikChange II XL kit (Stratagene) by following the manufacturer's instructions. Plasmid pJCL87 was derived from pDR111 and contains a chloramphenicol resistance cassette and the IPTG inducible P_hyperspank_ promoter. Plasmid pMG30 was constructed from pJCL87 by cloning the first 330 bp of the *map* gene under the control of the P_hyperspank_ promoter.

### Construction of strains

All strains used in this study are derived from the wild type strain JH642 (RB247) and listed in Table 1. The construction of strain RB301 and RB418 has been described previously [16]. RB395 was constructed by transforming RB247 with pMA1 and knocking out the native *rbgA* gene by using a MLS cassette. Strain RB1006 was constructed by transforming RB247 with plasmid pMG28 at the *amyE* locus and knocking out the native *rbgA* gene by using a MLS cassette. The strains were checked for interruption of *amyE* by growth on starch plates. Strain RB1043 was constructed by transforming RB247 with plasmid pMG29 and knocking out the native *rbgA* gene by using chromosomal DNA from RB395. Independently, strain RB1044 was constructed in a manner identical to RB1043 to serve as a biological duplicate. All strains discussed in this study were confirmed for desired change using PCR to amplify the region of interest followed by sequencing.

### Suppressor screen

Strains RB1043 and RB1044 were used for suppressor analysis. A single colony from each of these strains was inoculated per flask (25 colonies per strain, total of 50 colonies) and grown at 37°C for 16 hours. The undiluted culture from each flask as well as two serial dilutions (10-, and 100-fold) were plated on LB plates and incubated overnight at 37°C. The parental strains RB1043 and RB1044 were also plated along with RB1006 carrying wild-type RbgA to serve as controls. Isolated colonies from eight strains-RB1051, RB1055, RB1057, RB1059 (from RB1043) and RB1061, RB1063, RB1065, and RB1068 (from RB1044) that grew faster than parental strains were identified and characterized further.

### Whole genome sequencing and bioinformatics analysis

Genomic DNA from RB247, RB1043, RB1051, RB1055, RB1057, RB1059, RB1061, RB1063, RB1065 and RB1068 was isolated using the Wizard genomic DNA isolation kit (Promega). The genomic DNA was analyzed on a 0.8% agarose gel to ensure that the quality was suitable for sequencing. Whole genome sequencing was performed on a Genome Analyzer II instrument equipped with a paired end module (Illumina) at the MSU Research Technology Support Facility. The sequencing reads obtained were quality tested using FASTQC and trimmed if needed. Next we aligned sequence reads from RB247 and RB1043 against the reference *B. subtilis* strain 168 genome using R2R software. We identified the insertion of pMG29 in RB1043 when compared with RB247 reads and the insertion of the MLS cassette in RB1043 at the native *rbgA* locus. The sequence of suppressor strains RB1051, RB1055, RB1057, RB1059, RB1063, RB1065 and RB1068 was then compared to RB1043 (the parental strain). In addition to the expected insertions found in RB1043 and each suppressor strain (corresponding to pMG28 at the *amyE* locus and the MLS cassette at the native *rbgA* locus) we identified only a single change in each suppressor strain in the *rplF* gene. The suppressor mutations that were identified utilizing the R2R platform were confirmed by PCR amplification of the *rplF* gene and sequencing the amplified product.

### Structure analysis

Homology model of L6 from *B. subtilis* was obtained by using Modeller 9.12 [48], utilizing the crystal structure of L6 (PDB code: 1RL6) from *G. stearothermophilus* as a template. Out of 20 models constructed, the model with lowest energy (molpdf) was chosen for further analysis. All structural analysis for figure 9 were carried out in Chimera using the 50S structure (PDB: 2AW4) [48], [49].

### Constructions of strains for determining the phenotype of the L6 protein in a wild-type background

Strain RB1095 was constructed by transforming RB247 with pMG30 such that that the expression of the *map* gene (at the end of the operon that contains the *rplF* gene) was placed under the control of the IPTG inducible P_hyperspank_ promoter. RB1102 was constructed by transforming suppressor strain RB1051 with chromosomal DNA from RB1095 and selecting cells on IPTG, chloramphenicol and MLS (lincomycin and erythromycin) such that the *rbgA*-F6A gene at *amyE* locus was selected and the mutated *rplF* gene operon was linked to the chloramphenicol marker. RB1103, RB1106 and RB1107 were constructed similarly by using RB1055, RB1065 and RB1068 as the parental strains, respectively. RB1117 was constructed by transforming RB247 with chromosomal DNA from RB1102 and selecting cells on IPTG and chloramphenicol, thus ensuring that this strain had a wild type *rbgA* gene at the native locus and the mutated *rplF* gene (operon was tagged with the chloramphenicol marker). RB1118, RB1121 and RB1122 were constructed similarly by utilizing chromosomal DNA from RB1103, RB1106 and RB1107 respectively. RB1123 was constructed by growing RB1117 on LB plates without chloramphenicol and IPTG such that the plasmid pMG30 was excised out leaving the mutated *rplF* gene in a wild type background. RB1125, RB1131and RB1133 were constructed similarly from RB1118, RB1121 and RB1122 respectively.

### Analysis of ribosome profiles and ribosome complexes

Ribosomal subunits were prepared by sucrose density centrifugation. 50S and 45S complexes were isolated from lysates of RB418 and RB301 cells, respectively as previously described [34]. RB1051, RB1055, RB1057, RB1063, RB1065 and RB1068 were grown to OD_600_ of 0.5 at 37°C in LB medium. Chloramphenicol (Sigma) was added to a final concentration of 100 µg ml^−1^ 5 minutes prior to harvest. Cells were harvested by centrifugation at 5000 g for 10 min and resuspended in lysis buffer [10 mMTris-HCl (pH 7.5), 60 mMKCl, 10 mM MgCl_2_, 0.5% Tween 20, 1 mM DTT, 1× Complete EDTA-free protease inhibitors (Roche) and 10 U ml^−1^RNase-free DNase (Roche)]. Cells were lysed by three consecutive passes through a French press set at 1400 to 1600 psi and clarified by centrifugation at 16000×g for 20 minutes. Clarified cell lysates were loaded on top of 10–25% sucrose density gradients equilibrated in buffer B (10 mMTris-HCl, pH 7.6, 10 mM MgCl_2_, 50 mM NH_4_Cl) and centrifuged using a SureSpin 630 rotor (Sorvall) for 4.5 hours at 30,000 rpm. Gradients were then fractionated on a BioLogic LP chromatography system (BioRad) by monitoring UV absorbance at 254 nm. Fractions corresponding to ribosomal subunits of interest were pooled, concentrated using 100 kDa cutoff filters (Millipore) and stored in buffer A (10 mM Tris-HCl, pH 7.6, 10 mM MgCl_2_, 60 mM KCl and 1 mM DTT) at −80°C. For qMS, we followed the protocol as described above except that we used 18–43% sucrose gradient that was centrifuged at 21000 rpm for 14 hours.

### *In vitro* maturation

Cell lysates from RB1043 and RB1055 were obtained as described above. Lysates were concentrated using 4 mL Amicon ultra-4 centrifugal filters with 10 kDa cutoff (Millipore). An equal volume of lysate was incubated at 37°C or 0°C for 1 hour, then loaded onto 18–43% sucrose gradient made in buffer C (10 mM Tris-HCl, pH 7.6, 20 mM MgCl_2_, 50 mM NH_4_Cl) followed by centrifugation at ∼82000 g for 14 hours at 4°C in SureSpin 630 rotor (Sorvall). Gradients were fractionated on BioLogic LP system (BioRad) monitoring absorbance at 254 nm.

### GTPase activity

The assay was performed as described [34]. Briefly, for measuring GTPase activity in the presence of ribosomal subunits/intermediates 100 nM RbgA protein was incubated with 100 nM 50S subunit or 45S subunit or 44S subunit and 200 µM GTP at 37°C for 30 minutes and for measuring intrinsic GTPase activity 2 µM RbgA protein was incubated with 200 µM GTP at 37°C for 30 minutes. We predetermined that under these conditions the values were in the linear range of the assay. The phosphate released was measured using the Malachite Green Phosphate Assay Kit (BioAssaySystems).

### Quantitative mass spectrometry

Experimental samples were prepared for quantitative mass spectrometry as described previously [22], with the following noteworthy modifications. First, each ^14^N-labeled sample to be analyzed (20 pmol) was mixed with a “double spike” internal reference standard mixture of ^14^N (10 pmol) and ^15^N (30 pmol) labeled 70S particles. The addition of the ^14^N standard still allowed for independent quantitation of the ^15^N isotope distribution for each peptide, but simultaneously ensured that each ^15^N peak bore a corresponding ^14^N peak pair irrespective of the concentration in the experimental sample. After precipitation, reduction, alkylation and tryptic digestion, peptides were analyzed on an Agilent G1969A ESI-TOF mass spectrometer. ^14^N:^15^N peak pairs were identified in the raw data, assigned to unique ribosomal peptides using a theoretical digest and the quantities of ^14^N and ^15^N species were calculated by fitting each isotope envelope using a Least Square Fourier Transform Convolution algorithm [50].

The contribution of the ^14^N material in the reference spike was eliminated from each experimental measurement by first analyzing the double spike mixture in isolation (Figure S2A; red). To account for variations in sample preparation and ionization efficiency between experiments, the ^14^N fitted amplitude of each peptide in each sample was normalized using the ^15^N internal standard amplitude (derived from a fixed concentration of ^15^N 70S ribosomes in each sample). Once normalized, the contribution of the double spike to the measured ^14^N amplitude could be eliminated from each experimental sample by simple subtraction of the ^14^N amplitude of the double spike sample measured in isolation (Figure S2C).

To test the efficacy of the approach and to establish a detection limit, we first measured a standard curve using 0, 2, 4, 8, 16, or 32 pmol of ^14^N-labeled 70S ribosomes mixed with the double spike (10 pmol ^14^N, 30 pmol ^15^N 70S). As our analysis pipeline depends on the identification of peak pairs, this double spike approach greatly increased the number of peptides detected for low-abundance proteins (J.H. Davis unpublished observation). Indeed, we consistently identified multiple peptides for each ribosomal protein, even at the low end of the standard curve (Figure S2D). By comparing the measured ^14^N/^15^N ratio to the known ratio added we found the approach to be linear over the range of this standard curve (Figure S2E). Moreover, this experiment established a quantitation limit of 2 pmol of the experimental sample, corresponding to occupancy = 0.1 when 20 pmol of the 44/45S or 70S particles were analyzed.

For each experimental sample, relative protein levels were calculated as the ^14^N corrected isotope distribution amplitude divided by the ^15^N isotope distribution amplitude. Isotope distributions and local chromatographic contour maps were examined and peptides with low signal-to-noise were excluded. Finally, to account for differences in the total amount of sample added, each relative protein level was normalized to that of the primary binding protein L20, which did not vary in occupancy between samples.

### Selective reaction monitoring

To improve our quantitation accuracy in more complex samples such as the cell lysates, we developed a selective reaction monitoring (SRM) protocol focused on ribosomal proteins. First, ^14^N-labeled tryptic peptides were generated from 70S particles as described above. These peptides were eluted from an analytical C18 nano-column across a 90 min concave 5–50% acetonitrile gradient at 300 nL/min. Mass spectrometry was performed on an AB/Sciex 5600 Triple-TOF instrument with an information dependent acquisition method utilizing 250 ms MS^1^ scans followed by 20 successive MS^2^ scans, each lasting 50 ms (cycle time of 1.3 sec, 4150 cycles/run). Each precursor was excluded from the MS^2^ target list for 12 seconds after observation. Using the fragmentation data and a theoretical digest of the *B. subtilis* proteome, precursor peptides were assigned using Mascot (Matrix Science). After generating a spectral library from the Mascot identifications, 8 SRM methods each targeting ∼110 ribosomal precursor ions each were generated in Skyline [51]. An equal mixture of ^14^N and ^15^N labeled peptides derived from 70S ribosomes were analyzed using these methods on the Triple-TOF and transitions with low signal-to-noise were eliminated. Using the measured retention times bracketed by a 7.5 min window, Skyline was used to generate a single scheduled MRM method targeting 310 precursors and ∼10 product ions per precursor. MS^1^ and MS^2^ scans lasted 200 ms and 30 ms, respectively. To measure ribosomal protein levels in cellular lysates, 0.5 OD*mL of each culture was mixed with 20 pmol ^15^N-labeled 70S ribosomes and prepared for qMS as described above. Each sample was then analyzed using the scheduled MRM method. Transition chromatograms were extracted from the raw data using Skyline and ^14^N/^15^N peak areas were calculated, filtered to exclude those with low signal-to-noise, and plotted using a series of Python scripts.

The Pearson correlation coefficient, r, was calculated between the whole cell protein level and immature particle datasets for strains RB1043, RB1055, RB1057, and RB1068 using the SciPy library.

## Supporting Information

Figure S1 A. Multiple sequence alignment of L6 protein from selected bacterial species. B. Multiple sequence alignment of L6 protein from selected archaeal species. C. Multiple sequence alignment of L9 (a homologue of bacterial L6) protein from selected eukaryotic species. Substitutions in *rplF* partially suppress the growth defect of *rbgA*-F6A. The positions of the mutated residues are highlighted in yellow. Alignments were constructed with ClustalW with default parameters and species indicated to the left. ‘*’ indicates positions which have a single, fully conserved residue, ‘:’ indicates conservation between groups of strongly similar physicochemical properties, ‘.’ indicates conservation between groups of weakly similar physicochemical properties.
(TIF)

Figure S2 Characterization of a SILAC-like qMS protocol. **A**. Extracted mass spectra corresponding to an L16 peptide [residues 88–101]. ^14^N spectra (left) resulting from the addition of 0 (red), 2 (blue), 4 (green), 8 (purple), 16 (orange), or 32 (grey) pmol 70S particles to a mixed spike of 10 pmol ^14^N+30 pmol ^15^N 70S particles. **B**. Each isotope distribution is fit (see materials and methods) and normalized to the fit amplitude of the corresponding ^15^N peak. **C**. Once normalized, the contribution of the reference spike to the ^14^N amplitude is eliminated by subtracting the spike alone ^14^N amplitude (red) from each sample. **D**. Peptide coverage for large subunit proteins. Each semitransparent dot represents one measurement. Median measurement values are shown with a larger opaque marker. Values are reported as the ^14^N to ^15^N fitted amplitude ratio, after subtraction of the ^14^N reference sample contribution. **E**. Correlation between ^14^N material added (x-axis) and measured ^14^N to ^15^N fitted amplitude ratio (y-axis). Each sample bears a fixed concentration of ^15^N peptides (30 pmol).
(TIF)

Figure S3 Measurement of GTPase activity of RbgA in the presence of 44S intermediate from suppressor strains. The intrinsic GTPase activity of RbgA (column 1) was determined by incubation of 2 µM RbgA protein with 200 µM GTP for 15 minutes at 37°C. Stimulation of GTPase activity was measured by incubation of 100 nM RbgA protein with 100 nM of mature 50S subunit (column 2); 45S complex isolated from RbgA depleted cells (column 3); 44S intermediate isolated from suppressor strain RB1051 (column 4); 44S intermediate isolated from suppressor strain RB1055 (column 5); 44S intermediate isolated from suppressor strain RB1057 (column 6); 44S intermediate isolated from suppressor strain RB1063 (column 7); 44S intermediate isolated from suppressor strain RB1065 (column 8) and 44S intermediate isolated from suppressor strain RB1068 (column 9). The values represent the average of three independent experiments and the error bars represent the S.D.
(TIF)

Figure S4 Mutations in L6 protein affect subunit joining/interaction. Ribosome profiles of strains expressing mutated L6 protein RB1125 (*rplF*-R3C, panel 2), RB1131 (*rplF*-G5S, panel 3) and RB1133 (*rplF*-T68R, panel 4) show a higher concentration of individual ribosomal subunits and lower concentration of 70S ribosomes compared with ribosome profile of wild-type cells (panel 1). The X-axis indicates the direction of the profiles from the bottom of the gradient (25%) to the top of the gradient (10%). The Y-axis depicts absorbance at 260 nm, which is equivalent for all plots depicted. Dashed lines indicate the position of the 70S, 50S and the 30S complexes in the gradient.
(TIF)

Figure S5 Whole cell protein abundance full dataset. The full dataset used to derive Figure 7. Protein abundance is calculated as the ^14^N/^15^N ratio and is normalized to that of L20 for each sample. In contrast to Figure 7, plotted values are not normalized to RB301. Datapoints are colored and proteins are labeled as in 8.
(TIF)

Figure S6 L6-h97 (ScL9-h97) interactions are conserved from bacteria to eukaryotes. **A**. Crystal structure of 60S subunit from *Saccharomyces cerevisiae* (PDB ID: 3U5D and 3U5E) with the positions of ribosomal protein L9 (ScL9, homolog of bacterial ribosomal protein L6, cyan) and ribosomal protein L10 (homolog of bacterial ribosomal protein L16, green) highlighted. **B**. A magnified view of the interaction between ScL9 (cyan) and h97 (magenta) is shown. *E. coli* L6 (indicated as EcL6 in blue) from 50S structure (PDB ID: 2AW4) is superimposed on ScL9 and corresponding residues mutated in *Bacillus subtilis* suppressor strains are shown in red.
(TIF)
