# Supplementary figures and images for: Functional Interaction between Ribosomal Protein L6 and RbgA during Ribosome Assembly
Source: PLoS Genet. 2014 Oct 16;10(10):e1004694. doi: 10.1371/journal.pgen.1004694 (PMC4199504; doi:10.1371/journal.pgen.1004694)

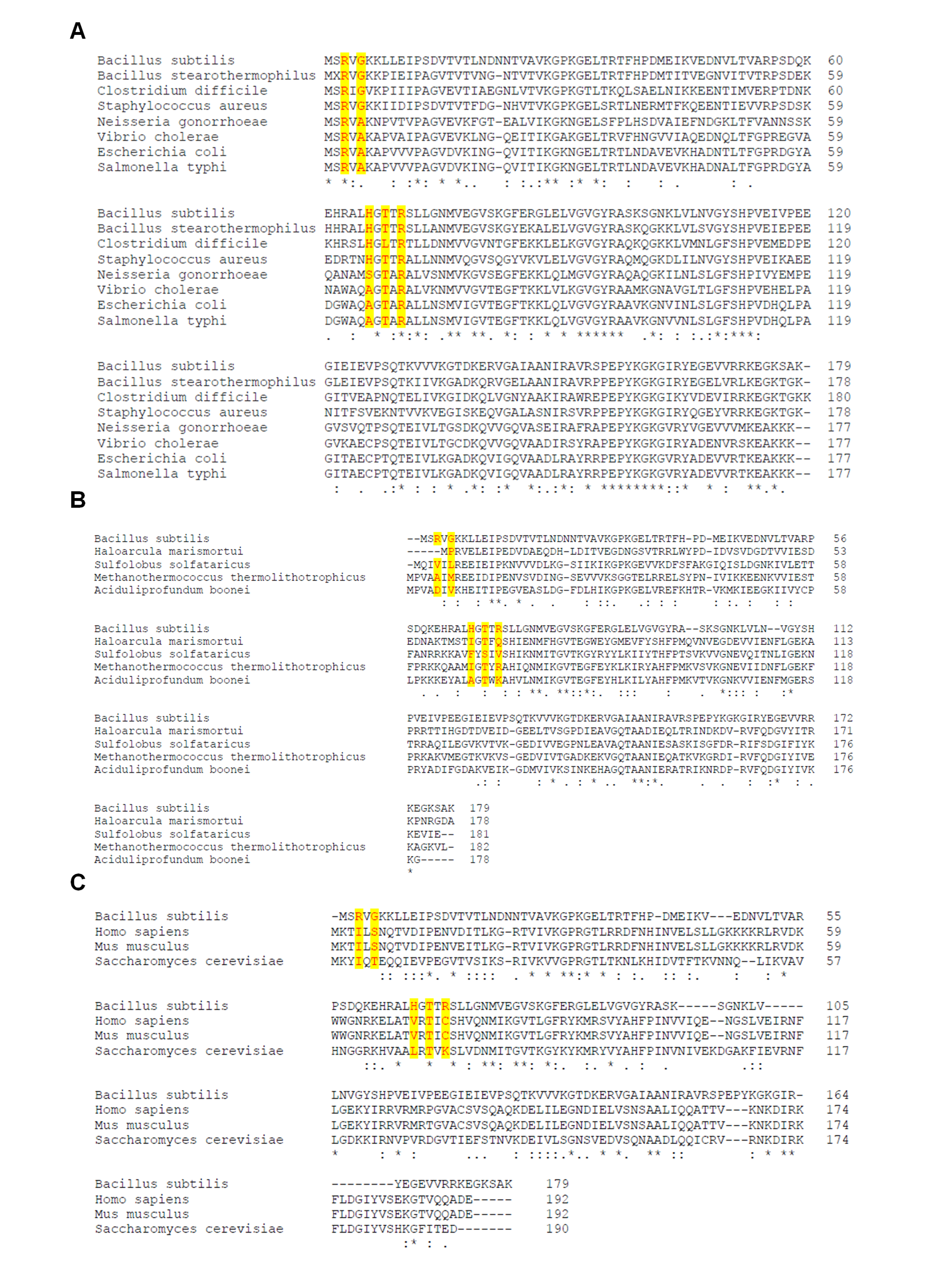

Supplement: Figure S1 — A. Multiple sequence alignment of L6 protein from selected bacterial species. B. Multiple sequence alignment of L6 protein from selected archaeal species. C. Multiple sequence alignment of L9 (a homologue of bacterial L6) protein from selected eukaryotic species. Substitutions in rplF partially suppress the growth defect of rbgA-F6A. The positions of the mutated residues are highlighted in yellow. Alignments were constructed with ClustalW with default parameters and species indicated to the left. ‘*’ indicates positions which have a single, fully conserved residue, ‘:’ indicates conservation between groups of strongly similar physicochemical properties, ‘.’ indicates conservation between groups of weakly similar physicochemical properties. (TIF) [file pgen.1004694.s001.tif]

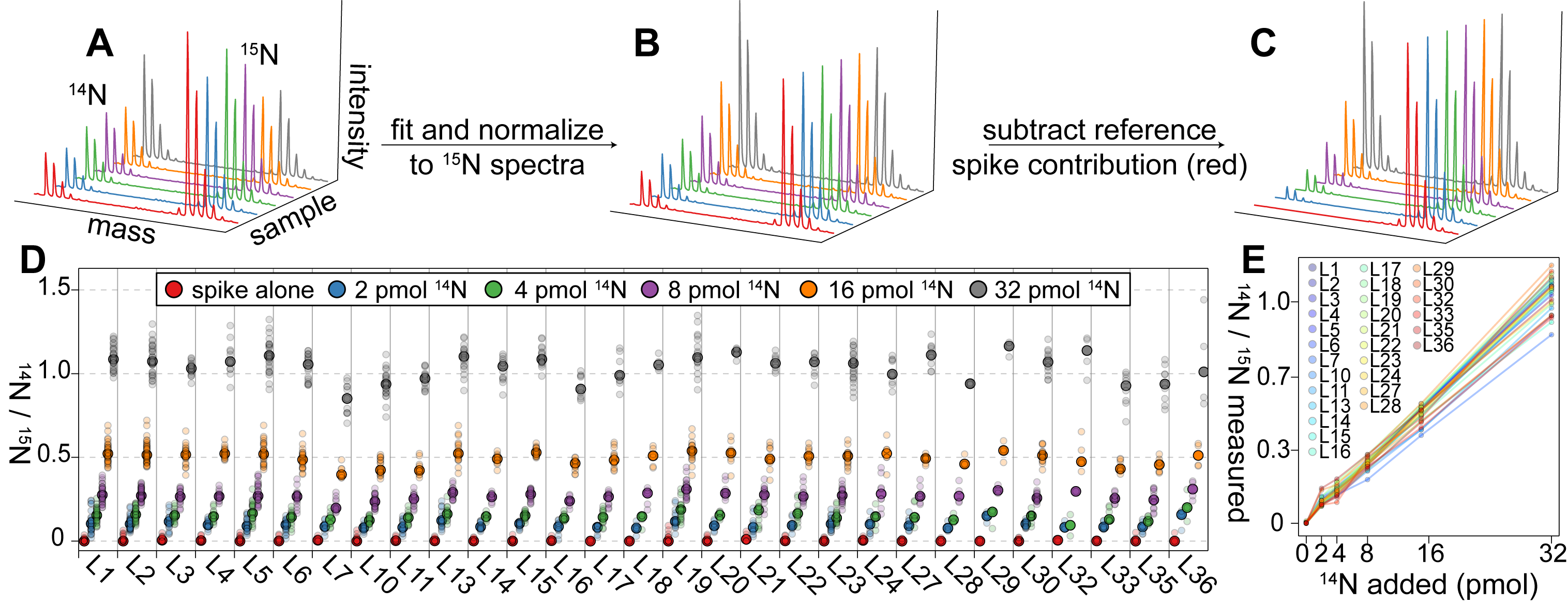

Supplement: Figure S2 — Characterization of a SILAC-like qMS protocol. A. Extracted mass spectra corresponding to an L16 peptide [residues 88–101]. 14N spectra (left) resulting from the addition of 0 (red), 2 (blue), 4 (green), 8 (purple), 16 (orange), or 32 (grey) pmol 70S particles to a mixed spike of 10 pmol 14N+30 pmol 15N 70S particles. B. Each isotope distribution is fit (see materials and methods) and normalized to the fit amplitude of the corresponding 15N peak. C. Once normalized, the contribution of the reference spike to the 14N amplitude is eliminated by subtracting the spike alone 14N amplitude (red) from each sample. D. Peptide coverage for large subunit proteins. Each semitransparent dot represents one measurement. Median measurement values are shown with a larger opaque marker. Values are reported as the 14N to 15N fitted amplitude ratio, after subtraction of the 14N reference sample contribution. E. Correlation between 14N material added (x-axis) and measured 14N to 15N fitted amplitude ratio (y-axis). Each sample bears a fixed concentration of 15N peptides (30 pmol). (TIF) [file pgen.1004694.s002.tif]

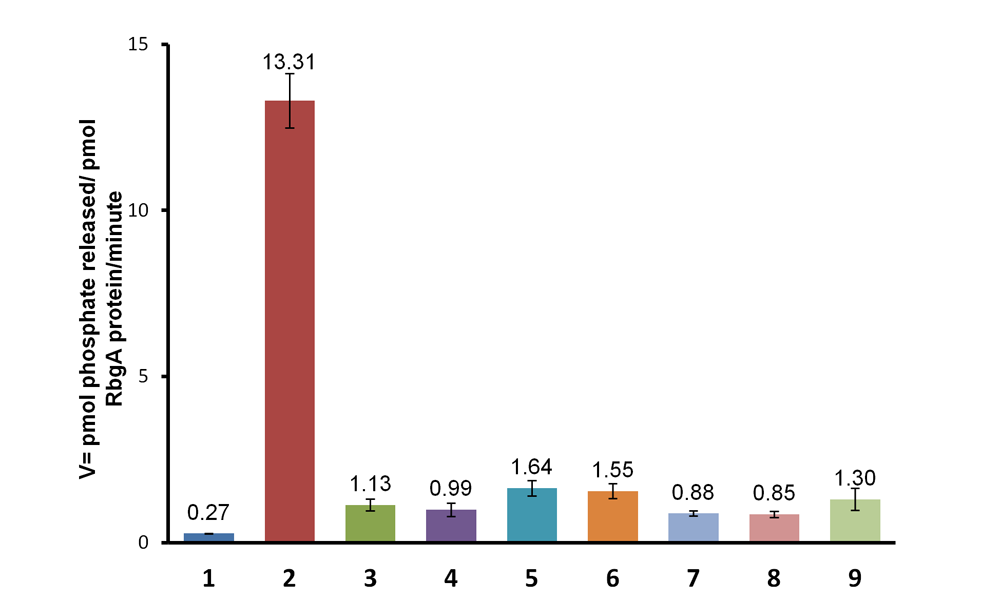

Supplement: Figure S3 — Measurement of GTPase activity of RbgA in the presence of 44S intermediate from suppressor strains. The intrinsic GTPase activity of RbgA (column 1) was determined by incubation of 2 µM RbgA protein with 200 µM GTP for 15 minutes at 37°C. Stimulation of GTPase activity was measured by incubation of 100 nM RbgA protein with 100 nM of mature 50S subunit (column 2); 45S complex isolated from RbgA depleted cells (column 3); 44S intermediate isolated from suppressor strain RB1051 (column 4); 44S intermediate isolated from suppressor strain RB1055 (column 5); 44S intermediate isolated from suppressor strain RB1057 (column 6); 44S intermediate isolated from suppressor strain RB1063 (column 7); 44S intermediate isolated from suppressor strain RB1065 (column 8) and 44S intermediate isolated from suppressor strain RB1068 (column 9). The values represent the average of three independent experiments and the error bars represent the S.D. (TIF) [file pgen.1004694.s003.tif]

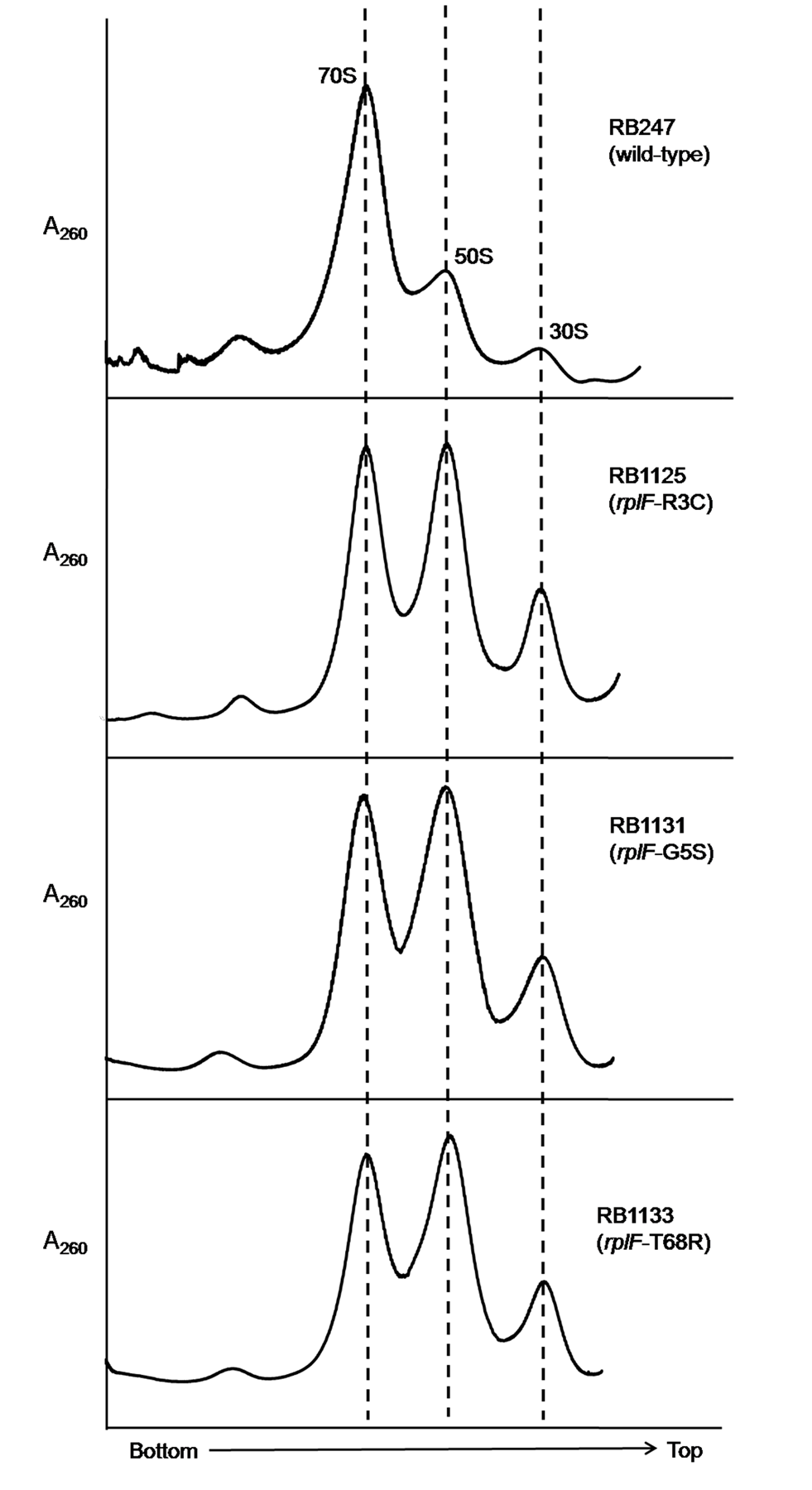

Supplement: Figure S4 — Mutations in L6 protein affect subunit joining/interaction. Ribosome profiles of strains expressing mutated L6 protein RB1125 (rplF-R3C, panel 2), RB1131 (rplF-G5S, panel 3) and RB1133 (rplF-T68R, panel 4) show a higher concentration of individual ribosomal subunits and lower concentration of 70S ribosomes compared with ribosome profile of wild-type cells (panel 1). The X-axis indicates the direction of the profiles from the bottom of the gradient (25%) to the top of the gradient (10%). The Y-axis depicts absorbance at 260 nm, which is equivalent for all plots depicted. Dashed lines indicate the position of the 70S, 50S and the 30S complexes in the gradient. (TIF) [file pgen.1004694.s004.tif]

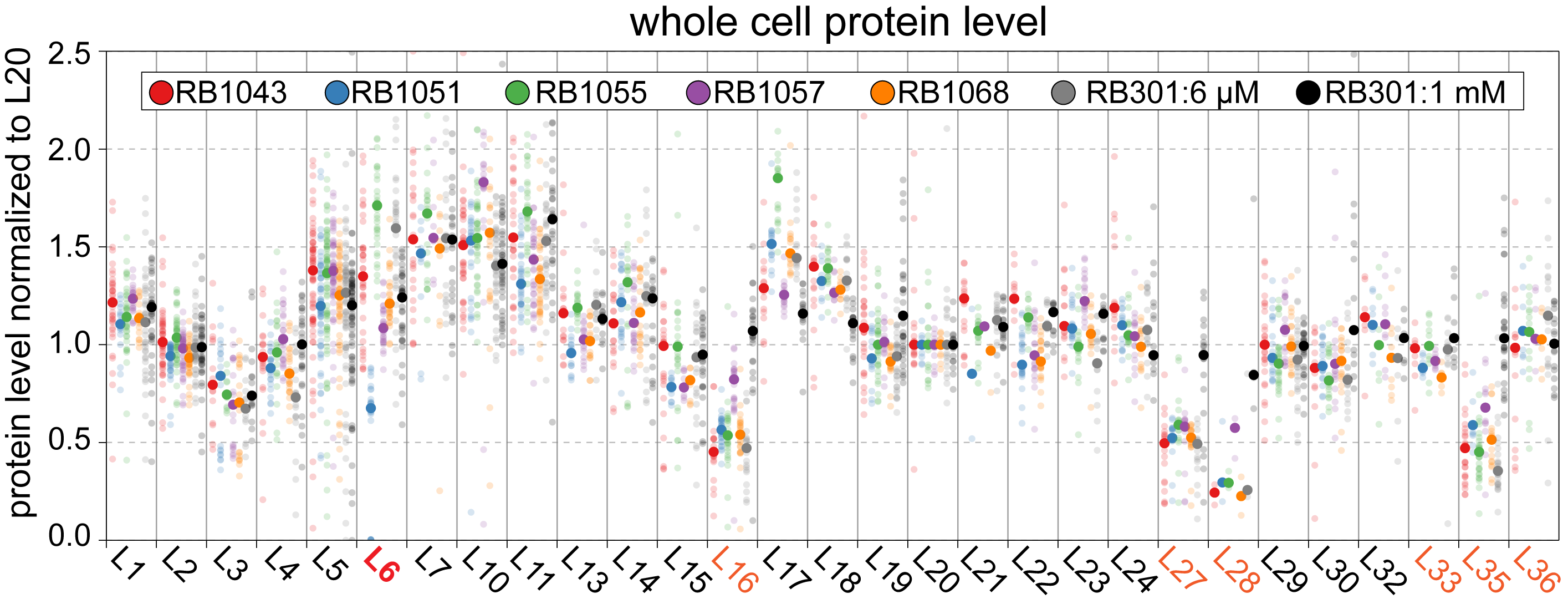

Supplement: Figure S5 — Whole cell protein abundance full dataset. The full dataset used to derive Figure 7. Protein abundance is calculated as the 14N/15N ratio and is normalized to that of L20 for each sample. In contrast to Figure 7, plotted values are not normalized to RB301. Datapoints are colored and proteins are labeled as in 8. (TIF) [file pgen.1004694.s005.tif]

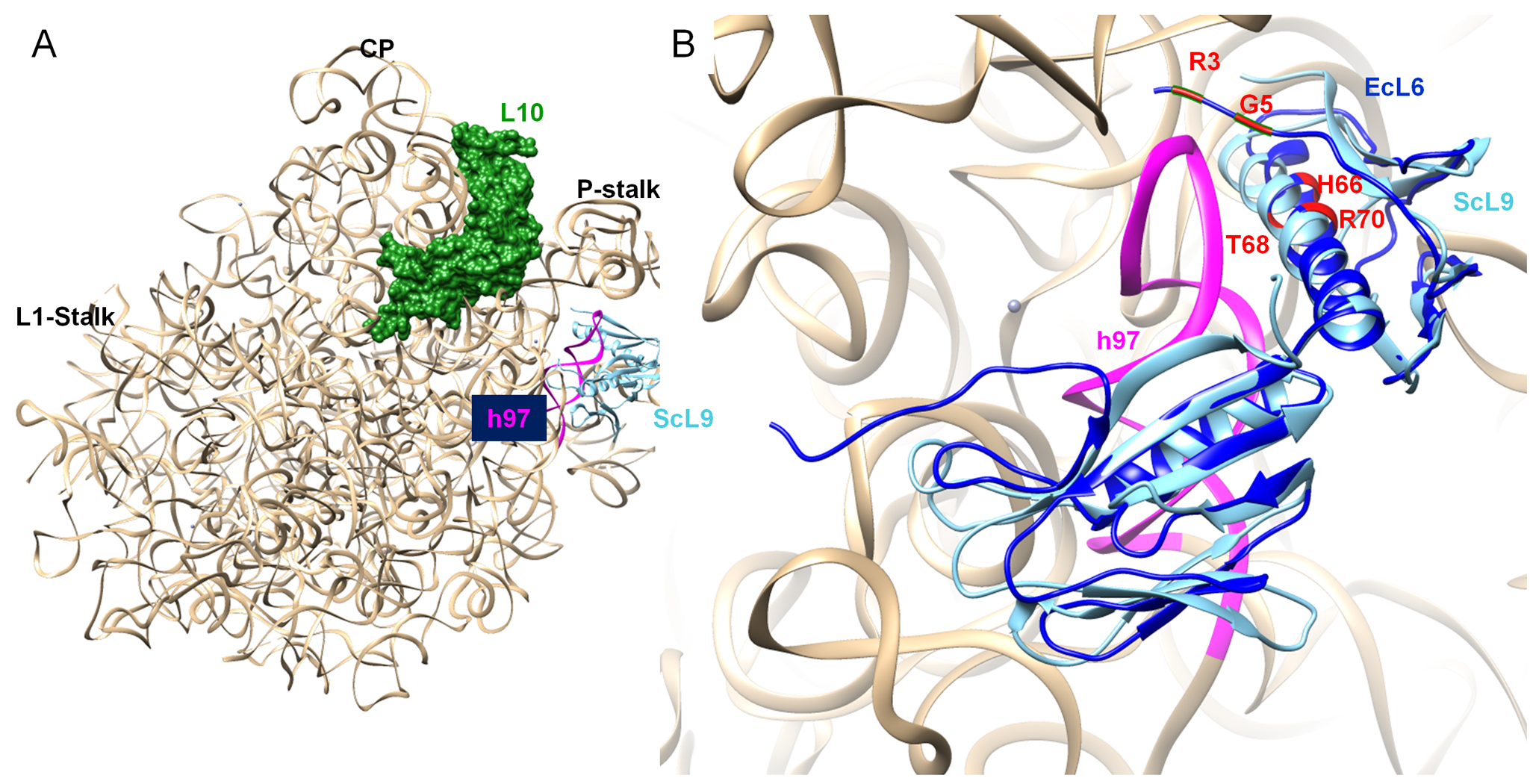

Supplement: Figure S6 — L6-h97 (ScL9-h97) interactions are conserved from bacteria to eukaryotes. A. Crystal structure of 60S subunit from Saccharomyces cerevisiae (PDB ID: 3U5D and 3U5E) with the positions of ribosomal protein L9 (ScL9, homolog of bacterial ribosomal protein L6, cyan) and ribosomal protein L10 (homolog of bacterial ribosomal protein L16, green) highlighted. B. A magnified view of the interaction between ScL9 (cyan) and h97 (magenta) is shown. E. coli L6 (indicated as EcL6 in blue) from 50S structure (PDB ID: 2AW4) is superimposed on ScL9 and corresponding residues mutated in Bacillus subtilis suppressor strains are shown in red. (TIF) [file pgen.1004694.s006.tif]
